# Experiments

**Published:** 1800-08

**Authors:** Charles Darwin


### *Experiments, by Dr.* CHARLES DARWIN.

[ Continued from p. 50—53 of our laft. ]

EXP. XV. Two ounces of vifcid *mucus*, blown from the nofe, were put into a glafs; to thefe were added two drachms of *alkaline lixivium*; a *complete folution* foon took place; and upon the addition of two oz. of water, there did not occur any feparation. The glafs was allowed to remain at reft for the fpace of a fortnight, and during that time a very flight feparation only had taken place; which confifted folely of light flakes of mucus fwimming in the water; but nothing appeared in the bottom of the glafs, which could in the leaft degree deferve the name of fediment. Previous to making this and the preceding experiments, it is neceffary to examine the water to be ufed, as I once was furprized to fee a confiderable cloud, and then a fediment occur on adding water to mucus and alkaline lixivium; but I foon difcovered that this was owing to the impurity of the water added. Alfo in conducting this experiment, we muft let the mucus and the alkaline lixivium remain at leaft an hour united, before we add the water; and if we obferve any mucus undiffolved by the alkali, it fhould be removed before we add the water.

N. B. In fome repetitions of thefe experiments, I have feen a flight cloud in the fpace of twelve hours, but never immediately.

EXP. XVI. Two drachms of *purulent matter*, obtained in the opening of a large abfcefs, were put into a glafs, and agitated with about as much of the *alkaline cauftic* lixivium; in a fhort time there was an intimate mixture, and the pus feemed to be diffolved. But upon the addition of water a feparation took place, and in a few minutes a confiderable fediment, apparently of purulent matter, funk to the bottom. When allowed to remain at reft, it continued in this condition for feveral days: But at the end of a fortnight, what had at firft appeared as a fediment floated in the furrounding fluid.

N. B. It will fometimes be difficult to unite the pus and alkali.

[ To be continued. ]

